# Transactions of the American Medical Association, Vol. IX. 1856

**Published:** 1857-04

**Authors:** 


### BOOK NOTICES.

*Transactions of the American Medical Association, Vol. IX.* 1856. Philadelphia: Printed for the Association by T. R. & P. G. Collins.

(Continued from March No.)

The next paper in the Volume of Transactions, is a 'Report on the Sanitary Police of Cities,' by James M. Newman, M.D. of Buffalo, N.Y.

This is a well-written and interesting paper, occupying fifty pages. It contains statistical tables, showing the ratio of annual mortality in Boston, New York, Philadelphia, Baltimore, Charleston, New Orleans, and Chicago. A comparison is also made between the mortality of cities and rural districts, showing the great preponderance of deaths from certain diseases in the former. In addition to the statistical part of the Report, the writer discusses with ability the subjects of good water, pure air, sewerage, paving, &c. and closes with an appendix, showing the condition and extent of *Tenement Houses* in New York and Brooklyn.

We come next to a 'Report on the Treatment of Cholera Infantum,' by A. J. Fuller, M.D. of Bath, Maine.

This Report occupies only six or seven pages of the ponderous volume before us. But, short as it is, it might have been made much shorter, for the whole of Dr. Fuller's pathology and indications for treatment are contained in the following brief sentences:—

'From the fact of the occurrence of the disease at the time of teething, when the muciperous glands are in a state of great functional excitability, it appears evident that cholera infantum is a disease seated in the mucous follicles of the intestines; and that the hepatic congestion *is necessarily connected with it; the disease of the follicles being a secondary affection, caused by the congested state of the Liver*, by which a free passage of blood is prevented.' * * * * 'The treatment should be mainly directed to the glandular congestion, on which the disease depends; for, if once a *free secretion of bile is obtained*, the disease may generally be considered as overcome.'

As might be expected from this pathology, the author relies in the early stage mostly on leeches to the right hypochondrium, and small doses of calomel; and, in the advanced stage, on the warm bath, stimulating frictions, mild astringents, and tonics. It is scarcely necessary to state that we regard both the pathology and the treatment as exceedingly defective.

The next paper is a 'Report on the Use and Effects of

Applications of Nitrate of Silver to the Throat, either in Local or General Disease,' by Horace Green, M.D. &c. of New York.

This paper occupies thirty-five pages, and presents an interesting summary of the author's well-known views in relation to the application of nitrate of silver to the larynx, trachea, and bronchial tubes. Although the views of Dr. Green have already been made known to the profession so extensively that a detailed analysis of this report is unnecessary, yet we quote below what he says in relation to direct injections into the bronchial tubes:—

'It was our intention, however, to have illustrated the great value of this therapeutic agent, in the treatment of the different forms of disease, to which we have referred by the history of cases which have fallen under our own observation, which would have corroborated fully the favorable reports made by the preceding authors: but this paper is already sufficiently extended. Justice to this subject, however, would not be done, should we fail to allude altogether to the success which, during the last eighteen months, has attended the still farther extension of topical medication, in the treatment of thoracic disease, effected by means of the operation of *catheterism* of the air-passages, or the injections of a solution of the nitrate of silver into the bronchial divisions.

'During the last eighteen months, or since October, 1854, over one hundred patients, embracing cases of both pulmonary and bronchial disease, have been treated by this form of topical medication, conjoined with appropriate general remedies. The history of this plan of treatment and the results of the practice, results which have been in a high degree satisfactory, have been brought before the profession in papers read before the New York Academy of Medicine; before the State Medical Society of New York; and, more recently, a detailed report, embracing a statistical table of one hundred and six cases, thus treated, was published in the pages of the *American Medical Monthly*. Besides their publication in this country, most of these papers have been reprinted in some of the medical publications of Great Britain, and have also been translated and republished in a few of the leading journals of France. It will, therefore, be unnecessary to bring the whole subject before the Association; and we shall close the present report by a brief analysis of the cases embraced in the statistical table, which, with the history of many of these cases, may be found in the *American Medical Monthly* for March, 1856.

'Of one hundred cases of thoracic disease treated by cathe-m of the air-passages, seventy-one of the sum total are ·ded as cases of *tuberculosis.* Of this number, *thirty-two* were considered cases of *advanced phthisis*—cases in which tubercular cavities were recognized in one or both lungs; and *thirty-nine* cases of *early phthisis.* Of the first division—advanced phthisis—fourteen have since died. *Twenty-five* were more or less improved; their lives being apparently prolonged by this method of medication. *Seven* only of the thirty-two cases of advanced phthisis were not benefited by the injections.

'Of the *thirty-nine* cases of incipient tuberculosis, twelve of this disease have apparently recovered. Five more of this number are now, or were, at the time of making the report, in the enjoyment of a good degree of health. With respect to the above twelve cases, I say *apparently* cured; for, although the appearance of these patients, as manifested both by the physical and rational signs, is indicative of an ordinary degree of health, yet, in a disease like that of tuberculosis, every medical man is aware that one year is a period too brief to speak decidedly with regard to the positive and final result.

'Of the remaining *twenty-two* cases, many of whom, at the time of the report, were still under treatment, *seventeen* had been greatly improved by topical medication; three more had been moderately benefited; while *three* only had failed to obtain any advantage from the local measures which had been adopted.

'Of the *twenty-eight* cases of *bronchitis,* sixteen had been dismissed cured, or so much improved as to require no further treatment. All the others had been greatly benefited, although some were still under treatment at the time of making the report.

'Finally, in view of all that has been accomplished by topical medication, the Chairman of the Committee would reiterate the declaration made in the first paper communicated to the professional public on this subject, that "the results of this method of treating disease, whether it has been employed in bronchial affections, or in the commencement of tuberculosis, have already afforded the most gratifying indications that practical medicine will be greatly advanced by this discovery."*'

The next twenty pages in the Transactions are occupied by a 'Report on the Best Mode of Rendering the Patronage of

* American Medical Monthly, Jan. 1855, p. 25.

the National Government Tributary to the Honor and Improvement of the Profession,' by Joshua B. Flint, M.D. of Ky.; and then comes the Report of the Standing Committee on Medical Education, by its Chairman, Wm. Henry Anderson, M.D of Alabama.

From the last-named paper we copy the following, and commend it to the careful consideration of such of our neighbors as have been trying for the last three or four years to make students believe, that clinical instruction in hospitals was of no value in connection with our medical colleges.

'The science of Medicine is such by its very nature, that it can be properly taught only in populous towns. The practical part of it ought to go on *pari passu* with the theoretical. If it do not, the graduate must go forth into the world incompetent to do his duty. If the population surrounding him could judge of his qualifications, all danger would be averted, and the evil would remedy itself. But inasmuch as the masses *must* be ignorant on those very subjects which most conduce to their welfare and happiness, they ought, if possible, to be protected by proper legislation. All educated physicians are aware that practical medicine can only be studied in hospitals and infirmaries; but the desire for popular applause, and thirst for reputation or a name, induce many to seek charters for colleges to be located in any little town or village in which they may happen to reside. The fault exists not in the fact that there are too many medical institutions, but that one-half of them have not the facilities to teach the science as it should be taught. The wants of our population require three thousand graduates yearly, and we believe that this number would be much better prepared at a dozen colleges, than at two or three; but those colleges should exist at such places as could afford sufficient clinical instruction, and, as it would be impossible to draw the line of demarcation which would constitute a city sufficiently populous for a medical college, there ought, in justice to the community, to be a higher tribunal before which all graduates should appear, and be impartially examined before receiving license to practice. If the licentiates of colleges from town and villages would in every case seek populous cities to perfect their education in hospitals and infirmaries, all would be well enough—but the *few* only do this, while the *many* go out into the world unprepared to do anything but injure the community, and degrade the profession by their inability and ignorance.'

The leading distinctive idea in the report of Dr. Anderson, however, consists in the recommendation that students of medicine become more thoroughly versed in the *elements* of each branch of medical science before attending any medical college. He says:—

'It is believed that three-fourths of the medical students who fill our numerous colleges, receive their earliest instruction in the rudiments of the profession in the offices of country physicians, and in small villages, where they get a very inadequate idea of any branch of medicine. If this large number of students could enter college well prepared, they might receive from two courses of lectures a very respectable share of medical knowledge. All will agree that the subjects embraced in a course of lectures include all that is necessary, and that the various chairs as a general rule are filled with competent professors: but the difficulty is that the students cannot comprehend the largest portion of the lecture, and even if they could, a vast amount of time is wasted on subjects that might be learned before entering college. If the student were well prepared, much of the professor's time might be expended on subjects of a more practical nature. Aware of this fact, a previous report to this Association recommended the establishment of preparatory schools. Much good was expected from the adoption of this plan; but experience has proved that as a general rule it could not be adopted, as it could only be put into operation in towns and cities, thereby effectually cutting off the great number of students from these useful institutions. The idea of a preparatory education was an excellent one, since it is from this source alone that we are to expect any radical change in our present system. How then is the candidate for admission into a medical college to become prepared to enter with advantage his collegiate course? This is a subject to which your reporter has directed his attention especially, and on which he has reflected a good deal.

'A great amount of matter which, under the present system, necessarily enters into a professor's lecture, might be learned in the office of the physician, or even in the closet of the student, before he commences a course of lectures. If, then, he can get an accurate knowledge of this large amount of medical matter, he could devote his collegiate period to a much more extended course of study than he could under other circumstances. The professor would not have to waste time on subjects purely elementary, but could plunge at once into important practical matters, which he is now debarred the satisfaction of doing, because he knows he cannot be thoroughly understood.'

To carry into effect his leading idea, the reporter recommends the following project:—

'The question now arises, From what source is information on all these subjects to be derived? It is true they are all discusssed in a clear and practical manner in books already published, but in such books they are so interspersed with other matters as to be of little value to the student who is just commencing his studies. Another book is needed; a book which will contain in one, or at most two volumes, dissertations on the various subjects which enter into the course of medicine. It would be no difficult task for an experienced member of the profession to prepare such a text-book, and there is little doubt that if it met the end proposed, it would have a circulation co-extensive with the *United States Dispensatory* itself. Such a book would find its way into every physician's office, and the four or five thousand students who are annually commencing medicine, would purchase it as the most valuable preparatory work they could possibly procure. It would enable them to learn everthing necessary for a thorough comprehension of the lectures delivered in any college in the United States, and would so lighten their collegiate labors that they might devote a large portion of time to those practical matters which, under the present system, must be neglected both for want of time on the part of the professor, and for want of capacity on the part of the student.'

The evil which the reporter here points out, and the object he wishes to have accomplished, are doubtless real and important; but we fear that he neither fully comprehends the one, nor sees clearly the only practicable method of accomplishing the other. That a large portion of students are not prepared to profit by some of the courses of lectures to which they listen in our colleges, is true. This arises, however, not from the want of a student's *vade mecum*, or book of elementary facts and principles in relation to all the branches of medicine, but simply because he is obliged to listen to all the branches at once, or during the same day; while, in truth, a thorough knowledge of some of them constitutes the only adequate preparation for entering profitably upon the study of the rest. For instance, the preparation which the student needs to enable him to listen profitably to courses of lectures on practical medicine, surgery, and midwifery, is not a mere smattering or superficial study of the outlines of these branches, but a thorough and detailed knowledge of anatomy, physiology, chemistry, and materia medica. The true remedy, then, is not in the study of any proposed *book*, or in making the courses of lectures in the colleges less elementary, but in so extending and dividing the annual college terms that each student can attend such a series of lectures as is suited to his particular period of advancement in his studies, without being obliged to include others for which he is wholly unprepared.

